# Parental Emotion Socialization and Child Adjustment in Greek Families: Supportive vs. Non-Supportive Parenting

**DOI:** 10.3390/children12070807

**Published:** 2025-06-20

**Authors:** Maria Markoulaki, Christina Dimitrakaki, Andoniki Naska, Katerina Papanikolaou, Georgios Giannakopoulos

**Affiliations:** 1 Department of Hygiene, Epidemiology and Medical Statistics, School of Medicine, National and Kapodistrian University of Athens, 115 27 Athens, Greece; mmarkoul@med.uoa.gr (M.M.); cdimitrakaki@med.uoa.gr (C.D.); anaska@med.uoa.gr (A.N.); 2 Department of Child Psychiatry, School of Medicine, National and Kapodistrian University of Athens, Aghia Sophia Children’s Hospital, 115 27 Athens, Greece; katpapan@med.uoa.gr

**Keywords:** children, parenting styles, emotional problems, behavior problems, social adjustment

## Abstract

**Background/Objectives**: Parental emotion socialization is a key influence on children’s emotional regulation and psychosocial development. This study examined how supportive and non-supportive parental responses to children’s negative emotions are associated with internalizing and externalizing problems and social competence among Greek children aged 6–12 years. **Methods**: A non-experimental, cross-sectional correlational study using convenience sampling was conducted with 100 Greek parents who completed the Coping with Children’s Negative Emotions Scale and the Child Behavior Checklist. Correlation and multiple regression analyses were used to examine associations between parenting responses and child outcomes, controlling for child age, child gender, and parent gender. **Results**: Minimization/devaluation responses were significantly associated with higher internalizing problems (β = 0.396, *p* = 0.009), externalizing problems (β = 0.264, *p* = 0.048), and total problems (β = 0.320, *p* = 0.012). Punitive responses significantly predicted externalizing (β = 0.383, *p* = 0.003) and total problems (β = 0.304, *p* = 0.004). Supportive strategies (e.g., emotion-focused and problem-focused responses) did not significantly predict lower problem scores but were positively correlated with social competence (e.g., problem-focused response: ρ = 0.25, *p* = 0.017). Parent gender predicted minimization/devaluation responses, with mothers scoring higher than fathers (β = 0.308, *p* = 0.006). **Conclusions**: Non-supportive parental responses—especially punitive and dismissive behaviors—are robustly associated with increased behavioral and emotional difficulties in children. While supportive strategies may contribute to social competence, their benefits appear diminished in the presence of negative parenting practices. These findings highlight the importance of culturally sensitive interventions tailored to Greek family dynamics. Despite limitations such as the use of self-report measures and a non-representative sample, this study contributes valuable insights into emotion socialization and child adjustment in a specific cultural context.

## 1. Introduction

Parental emotion socialization is a central construct in developmental psychology, reflecting the processes by which parents shape their children’s understanding, expression, and regulation of emotions [1,2,3]. Parental emotion socialization refers to the ways in which parents influence their children’s understanding, expression, and regulation of emotions through a variety of strategies, including modeling emotional responses, discussing emotions, and directly responding to children’s emotional expressions [2,4]. These socialization processes can be supportive—validating and guiding the child’s emotional experiences—or non-supportive—dismissing, punishing, or minimizing the child’s emotions. Over the past several decades, considerable attention has been given to how early family interactions contribute to the formation of emotional competence and overall psychosocial adjustment [5,6]. In this context, parental responses to children’s negative emotions—whether supportive or non-supportive—play a decisive role in influencing a child’s ability to manage stress, form healthy relationships, and navigate complex social environments [7,8,9,10]. For example, a supportive response might involve comforting a child who is upset and helping them find a solution to the problem, whereas a non-supportive response might include telling the child to stop crying or punishing them for showing distress.

Drawing on foundational theories such as attachment theory—which emphasizes the importance of early relational bonds in shaping later psychological functioning—researchers have argued that the ways in which parents respond to emotional distress provide critical models for children’s own emotion regulation strategies [11,12,13]. Supportive responses that involve acknowledging and validating a child’s emotional experience, guiding them through problem-solving, and encouraging adaptive coping tend to foster resilience and social competence [14,15,16,17]. Conversely, non-supportive responses, such as punitive or dismissive reactions, may predispose children to internalizing or externalizing difficulties by impairing their capacity to effectively manage negative emotions [18,19,20].

Despite these insights, several gaps remain in our understanding of parental emotion socialization, particularly when considering cultural and contextual factors. Much of the existing research has been conducted in North American or northern European settings, where cultural norms regarding emotional expression and family dynamics differ from those in other regions [21,22]. These studies have generally shown that supportive parenting practices, such as emotion coaching and validation, are associated with better emotional regulation and fewer behavioral problems in children, while non-supportive practices—like punishment or emotional dismissal—are linked to increased internalizing and externalizing symptoms. In Greece, traditional family values and cultural practices shape parenting styles in unique ways that may influence how emotion socialization unfolds in everyday life [23,24]. Traditional Greek families are typically characterized by close-knit family bonds, strong parental authority, and an emphasis on familial loyalty and emotional interdependence. Parenting practices often reflect a combination of protective behaviors and expectations of conformity, influenced by broader collectivistic cultural norms. Emotional expressiveness is valued within family settings, but children are also expected to demonstrate respect and self-regulation in their emotional displays. These cultural characteristics may shape the ways in which parents respond to children’s emotions, blending supportive strategies with more directive or controlling behaviors [23,24]. Recognizing these cultural nuances is essential, as they can moderate the relationship between parental behavior and children’s developmental outcomes [25,26,27].

From a lifespan developmental perspective, early experiences with emotion socialization are not isolated events; rather, they establish a foundation that influences emotional regulation and psychological well-being across different stages of life [28]. Research that integrates established theoretical frameworks with cultural considerations offers a more comprehensive understanding of how early family processes contribute to mental health outcomes over time. Focusing on a sample of parents of children aged 6 to 12 years—a critical period during which foundational emotional and social skills are rapidly developing—provides a valuable window into how early parental influences may shape a child’s trajectory toward adaptive functioning or increased vulnerability to psychological difficulties [29].

In summary, the present study is designed to address these gaps by examining the associations between parental emotion socialization and children’s psychosocial adjustment within a Greek sample. Specifically, it aims to determine whether supportive parental responses are associated with lower levels of emotional distress and behavioral challenges, whether non-supportive reactions are linked to elevated internalizing and externalizing problems, and how these associations are moderated by the unique cultural factors inherent in Greek parenting practices. By integrating theoretical models from attachment theory, emotion regulation, and cultural psychology, this research is expected to advance our theoretical understanding of emotion socialization and inform culturally sensitive interventions aimed at promoting supportive parenting practices and enhancing children’s long-term mental health outcomes.

Despite these insights, few studies have explicitly examined these dynamics within southern European or Mediterranean cultural settings, where emotional expressiveness and parental authority often co-exist. Previous findings suggest that both the attachment relationship and emotion regulation capacity may serve as key mechanisms linking parental emotion socialization with child adjustment [11,28]. However, little is known about how these pathways operate in Greek families. The current study addresses this gap by using a predictive analytic framework to examine associations between different types of parental responses and child outcomes, while also considering demographic factors such as parent and child gender.

Therefore, the primary goal of the present study was to investigate how different parental emotion socialization practices are associated with children’s psychosocial adjustment in a sample of Greek families. To guide this investigation, we posed the following research questions: (1) Are supportive parental responses (e.g., problem-focused, emotion-focused, expressive encouragement) associated with lower levels of internalizing and externalizing problems and higher levels of social competence in children? (2) Are non-supportive parental responses (e.g., punitive, minimization/devaluation) associated with higher levels of internalizing and externalizing problems? (3) Do these associations remain significant after accounting for child age, child gender, and parent gender? Based on existing literature, we formulated the following hypotheses: (1) Supportive parental responses (e.g., problem-focused, emotion-focused, expressive encouragement) would be associated with lower levels of internalizing and externalizing problems, and higher levels of social competence in children, possibly through enhanced emotion regulation. (2) Non-supportive parental responses (e.g., punitive, minimization/devaluation) would be associated with higher levels of internalizing and externalizing problems, potentially due to disruptions in the parent-child attachment dynamic or emotional invalidation. (3) These associations would remain significant even when controlling for child gender, child age, and parent gender, as examined through multiple regression analyses.

## 2. Materials and Methods

### 2.1. Research Design

This study employed a non-experimental, cross-sectional correlational design to examine the associations between parental emotion socialization and children’s psychosocial adjustment. Data were collected through self-report questionnaires administered to parents, allowing for the investigation of how different parental responses relate to internalizing and externalizing problems in children.

### 2.2. Participants

The sample comprised 100 parents of children aged between 6 and 12 years. The sample size was determined based on feasibility and in accordance with similar studies in this research area. While no formal power analysis was conducted, previous research examining emotion socialization and behavioral outcomes has used comparable sample sizes to detect medium effect sizes with sufficient statistical power. Each participating parent completed the questionnaires independently, reporting on only one child. No cases involved both parents completing measures for the same child, and no parent provided assessments for more than one child. Participants were recruited using convenience sampling from various urban areas in Greece, which may limit the generalizability of the findings. Inclusion criteria required that respondents be primary caregivers of a child within the specified age range and be proficient in Greek. Demographic characteristics, including age, gender, and other relevant background variables, are summarized in Table 1. Detailed socio-demographic information regarding parental education level, employment status, and perceived financial situation was not collected in this study. Similarly, data regarding the number of siblings and the child’s birth order were not gathered. Parents were not specifically asked about formal diagnoses (e.g., ADHD, ASD) for their children; however, based on general screening during recruitment, no major developmental concerns were reported by participating families.

### 2.3. Instruments

#### 2.3.1. Parental Emotion Socialization

Parental responses to children’s negative emotions were assessed using the Coping with Children’s Negative Emotions Scale (CCNES) [30]. This instrument evaluates multiple dimensions of parental behavior by classifying responses as either supportive (e.g., emotion-focused, problem-focused) or non-supportive (e.g., punitive, dismissive, minimizing). Example items from the CCNES include supportive responses such as helping the child understand their feelings or encouraging problem-solving (e.g., comforting the child when upset), and non-supportive responses such as reacting with frustration, ignoring the emotion, or dismissing the child’s feelings as exaggerated. These illustrative items help capture the spectrum of parental behaviors the scale assesses. The CCNES has demonstrated satisfactory reliability and validity in previous research [31]. In the present study, internal consistency for the CCNES subscales was as follows: Distress/Anxiety Response (α = 0.76), Punitive Response (α = 0.77), Expressive Encouragement Response (α = 0.93), Emotion-Focused Response (α = 0.91), Problem-Focused Response (α = 0.81), and Minimization/Devaluation Response (α = 0.87), indicating acceptable to excellent reliability.

#### 2.3.2. Children’s Psychosocial Adjustment

Children’s psychosocial functioning was measured using the Child Behavior Checklist (CBCL) [32,33]. The CBCL provides comprehensive assessments of internalizing (e.g., anxiety, depression) and externalizing (e.g., aggression, hyperactivity) problems, as well as overall social competence. Example items from the CBCL reflect a range of behavioral and emotional concerns, such as frequent crying, difficulty concentrating, disobedience at home, or challenges interacting with peers. These examples correspond to the internalizing, externalizing, and social adjustment domains measured by the instrument. The CBCL is widely recognized for its robust psychometric properties [34]. In the present study, internal consistency for the CBCL subscales was high, with Cronbach’s alpha values as follows: Internalizing Problems (α = 0.85), Externalizing Problems (α = 0.90), and Total Problems (α = 0.93), indicating excellent reliability in this sample. It should be noted that the CCNES has not been formally validated for use in Greek populations. Although it was translated and adapted with care, this represents a limitation that should be considered when interpreting the findings.

### 2.4. Procedure

Data collection was conducted over a two-month period. Potential participants were initially contacted through schools, community centers, and local parenting networks. Upon expressing interest, parents received comprehensive written and verbal explanations regarding the study’s purpose, procedures, and confidentiality protocols. A brief introductory session was then conducted by the first author to ensure that participants fully understood the questionnaires and to address any initial questions or concerns.

After this interaction, parents who agreed to participate provided written informed consent. In addition to completing the Coping with Children’s Negative Emotions Scale (CCNES) and the Child Behavior Checklist (CBCL), participants also supplied demographic information and data on potential confounding factors (i.e., parent’s gender and child’s age and gender). These supplementary data were collected to enhance the robustness of subsequent analyses by allowing for the control of these variables.

Participants had the option to complete the questionnaires in a quiet setting at home or in a controlled environment provided by the research team. During the administration of the questionnaires, the first author remained available to clarify any items if needed, thereby ensuring that all questions were properly understood and reducing the likelihood of response errors.

All responses were collected anonymously, with each questionnaire being assigned a unique code to protect participant confidentiality. The completed questionnaires and additional data were then securely stored for further analysis.

### 2.5. Ethical Considerations

This study was conducted in accordance with the ethical principles outlined in the Declaration of Helsinki. Ethical review and approval were waived because the research involved minimal risk and relied solely on the anonymous and voluntary participation of adult parents who completed standardized, non-invasive self-report questionnaires. No sensitive personal data or identifying information were collected, and no interventions or experimental procedures were conducted.

Prior to participation, all respondents were provided with detailed information about the study’s purpose, procedures, and confidentiality protocols. Written informed consent was obtained from all participants. Participation was entirely voluntary, and individuals were informed of their right to withdraw from the study at any time without any penalty. All data were collected and stored anonymously to ensure the privacy and confidentiality of participants.

### 2.6. Data Analysis

Descriptive statistics were computed to summarize participant demographics and scale scores. Prior to the main analyses, the distributions of all key variables were examined for normality. Variables exhibiting significant skewness were log-transformed to meet the assumptions of linear regression analyses, which were used in subsequent steps. For the correlation analyses, non-parametric Spearman’s rho was employed to assess associations between parental responses and children’s psychosocial outcomes. This approach was selected due to the ordinal nature of some response scales and the presence of residual skewness in several variables, even after transformation.

Multiple regression analyses were performed to assess the unique contributions of supportive and non-supportive parental responses in predicting internalizing and externalizing problems while controlling for potential confounding variables. Specifically, the following confounders were included in the models: child age was entered as a continuous variable, while child gender and parent gender were entered as categorical variables (with child gender coded as 0 for female and 1 for male, and parent gender coded as 0 for father and 1 for mother). Including these variables allowed us to account for their potential influence on the outcome measures.

For all regression models, standardized beta coefficients (β) were estimated and reported to facilitate comparisons of predictor effects. Statistical significance was determined at *p* < 0.05. Data analysis was performed using SPSS software (version 26.0).

## 3. Results

### 3.1. Descriptive Statistics of the Sample

A total of 100 Greek parents participated in the study; all were the biological parents of their children. Among the parents, 82.1% were mothers, and 55.7% of the children were boys. The average age of the children was 10 years (SD = 1.7). Detailed sample characteristics are provided in Table 1.

### 3.2. Descriptive Measures for CBCL and CCNES Scales

The Child Behavior Checklist (CBCL) was used to assess various aspects of children’s behavior. The mean score for internalizing problems was 5.33 (SD = 5.35) and for externalizing problems was 4.05 (SD = 3.72). The overall CBCL total problems score averaged 22.55 (SD = 4.29). The Coping with Children’s Negative Emotions Scale (CCNES) scores (range 1–7, with higher scores indicating more frequent use) were as follows: Distress/Anxiety Response 2.93 (SD = 0.68), Punitive Response 2.04 (SD = 0.63), Expressive Encouragement Response 5.05 (SD = 1.33), Emotion-Focused Response 5.53 (SD = 1.18), Problem-Focused Response 5.90 (SD = 0.82), and Minimization/Devaluation Response 3.07 (SD = 1.15). Table 2 provides additional descriptive details, including the observed minimum and maximum values and median scores.

### 3.3. Correlation Analyses

Spearman correlation coefficients were calculated between the CBCL and CCNES scales. The Distress/Anxiety Response was significantly and positively correlated with Anxiety/Depression (rho = 0.22, *p* = 0.047), Internalizing Problems (rho = 0.25, *p* = 0.021), and Social Problems (rho = 0.25, *p* = 0.023). The Punitive Response showed significant positive correlations with Aggressive Behavior (rho = 0.37, *p* = 0.001), Externalizing Problems (rho = 0.34, *p* = 0.001), and Total Problems (rho = 0.29, *p* = 0.006). The Expressive Encouragement Response correlated significantly with Activities (rho = 0.26, *p* = 0.017) and Overall Competence (rho = 0.27, *p* = 0.012). Additionally, the Emotion-Focused Response, Problem-Focused Response, and Minimization/Devaluation Response were each significantly correlated with various CBCL scales. Table 3 summarizes the complete set of Spearman correlations.

### 3.4. Regression Analyses

#### 3.4.1. Regression Analyses on CCNES Dimensions

Multiple linear regression analyses were performed with the CCNES dimensions as dependent variables and child gender, child age, and parent gender as independent variables. For the dimensions of Distress/Anxiety, Punitive, Expressive Encouragement, Emotion-Focused, and Problem-Focused responses, none of the demographic variables emerged as significant predictors. In contrast, the Minimization/Devaluation Response was significantly predicted by parent gender; mothers demonstrated higher scores compared to fathers (β = 0.132, *p* = 0.006). These findings are detailed in Table 4.

#### 3.4.2. Regression Analyses on CBCL Problems

Additional regressions were performed with CBCL Internalizing, Externalizing, and Total Problems as dependent variables. In these analyses, predictors included child gender, child age, parent gender, and the CCNES dimensions. Internalizing Problems were significantly predicted only by the Minimization/Devaluation Response (β = 0.135, *p* = 0.009). Externalizing Problems were significantly associated with both the Punitive Response (β = 0.206, *p* = 0.003) and the Minimization/Devaluation Response (β = 0.070, *p* = 0.048). Total Problems were significantly predicted by the Punitive Response (β = 0.186, *p* = 0.004) and the Minimization/Devaluation Response (β = 0.108, *p* = 0.012), with the latter showing a slightly greater standardized effect. These results are summarized in Table 5.

Overall, the findings highlight the distinct roles of supportive and non-supportive parental responses in child adjustment. Notably, punitive and minimization/devaluation behaviors emerged as the most consistent predictors of higher internalizing and externalizing symptoms, while supportive strategies correlated with social competence but did not significantly predict reduced problem behaviors. These results suggest that reducing negative parental reactions may be particularly critical in addressing behavioral and emotional difficulties in children.

## 4. Discussion

The present study contributes significantly to our understanding of how parental emotion socialization is associated with children’s psychosocial adjustment. The findings reveal that non-supportive parental responses—particularly punitive and minimization/devaluation behaviors—are strongly associated with increased internalizing and externalizing problems. Descriptive analyses indicated that while parents reported moderately high levels of supportive responses (e.g., emotion-focused and problem-focused) and lower scores on non-supportive dimensions (e.g., punitive responses), even these relatively lower frequencies of unsupportive reactions exert a substantial negative influence on child outcomes. This suggests that the detrimental impact of unsupportive practices may outweigh the benefits of supportive interactions, thereby highlighting a potential target for intervention.

One key interpretation of these results is that children who are frequently exposed to punitive or dismissive parental behaviors may not develop effective emotion regulation strategies. Over time, such non-supportive practices can lead to maladaptive coping patterns that predispose children to greater emotional distress and behavioral difficulties [18,19,35]. This is further supported by our regression analyses, which demonstrated that minimization/devaluation responses significantly predicted internalizing problems and, along with punitive responses, also predicted externalizing and total problems. In contrast, although supportive responses have been linked to enhanced emotional understanding and resilience [14,15,16,17], their influence may be overshadowed when even minimal non-supportive behaviors are present. This asymmetry underscores the critical need to minimize negative parental practices to promote healthier developmental outcomes.

The implications for practice are substantial. These findings advocate for intervention programs that prioritize the reduction of non-supportive parental responses [36,37]. Parent-training initiatives that incorporate emotion coaching and stress management techniques could enable caregivers to modify punitive behaviors and foster a more supportive home environment [5,38,39,40]. Notably, in the Greek cultural context—where traditional family dynamics and cultural norms strongly shape parenting practices—tailoring interventions to reflect these nuances may enhance their effectiveness and ultimately lead to improved mental health outcomes for children [25,26,27]. In the Greek context, parent-training initiatives might incorporate culturally relevant values—such as respect for authority and family cohesion—into emotion coaching strategies to enhance receptivity and engagement. Collaborations with schools and community centers could support broader implementation. Specifically, such intervention programs could integrate these familiar cultural values into evidence-based frameworks by helping parents recognize dismissive or punitive responses and practice supportive strategies aligned with Greek parenting norms. These programs could be delivered in accessible settings such as schools, pediatric clinics, and local community hubs. By tailoring approaches to local family dynamics, such initiatives would promote emotional communication within families and help reduce children’s emotional and behavioral difficulties.

These findings are consistent with previous cross-cultural research suggesting that parental emotion socialization practices are deeply influenced by cultural norms and values. For instance, studies in East Asian and Mediterranean societies—both characterized by collectivistic orientations—have found that emotional guidance and parental control often coexist, shaping emotion regulation in ways distinct from more individualistic contexts [21,23,24]. Similar to our findings in Greek families, punitive or dismissive responses in these settings are linked to adverse child outcomes, underscoring the potentially universal risk posed by non-supportive parenting. At the same time, the benefits of supportive responses may be moderated by cultural expectations around emotional restraint, hierarchical relationships, and relational harmony—factors that could attenuate their impact in more traditional family systems.

The observed associations between non-supportive parental responses and child difficulties align with the broader literature indicating that punitive or dismissive parenting practices are universal risk factors for emotional and behavioral problems [18,19,20]. Thus, while some of the results may seem expected, their replication in a culturally distinct sample contributes important cross-cultural validation to existing theoretical models.

Furthermore, our regression analyses revealed that among demographic variables, parent gender played a role in non-supportive responses, with mothers exhibiting higher levels of minimization/devaluation than fathers. This insight could guide the development of gender-sensitive training programs [41]. However, it is important to interpret this finding with caution, given the substantial imbalance in the number of mothers and fathers in our sample. The small number of participating fathers may limit the generalizability of gender-based comparisons. Nevertheless, this finding opens interesting avenues for further exploration. It may reflect gendered expectations in emotional caregiving or differences in stress exposure between mothers and fathers in Greek families. For instance, mothers may more frequently encounter children’s emotional expressions and feel pressured to manage them efficiently, sometimes leading to dismissive responses. Future studies with more balanced gender representation should examine whether these patterns hold and explore the underlying mechanisms.

Beyond practical applications, the study also enriches our theoretical understanding of emotion socialization. It supports the notion that early interactions within the family serve as a foundation for later emotional and behavioral regulation [5,6,28]. Importantly, the findings suggest that cultural context may moderate these relationships. In Greek families, for example, the interplay between traditional values and modern parenting practices might influence how parental responses affect children’s adjustment [23]. This observation invites further exploration into how cultural factors can both buffer and exacerbate the impact of parental behavior on child development.

It is important to acknowledge that the relationship between parental emotion socialization practices and children’s psychosocial adjustment is likely to be bidirectional. One plausible mechanism linking non-supportive parenting to child adjustment is the disruption of emotion regulation development, as children exposed to punitive responses may internalize maladaptive coping strategies [42]. From an attachment perspective, these parenting behaviors may also weaken the sense of emotional security needed for adaptive regulation [43,44]. Children’s emotional and behavioral characteristics, such as temperament, self-regulation skills, and even birth order, may influence how parents respond to their emotional expressions [45,46]. Furthermore, broader environmental factors, including socio-economic conditions, parental stress, and family structure, may simultaneously affect both parenting behaviors and child outcomes [47]. While the current study focused on parental responses as predictors, future longitudinal research incorporating assessments of child temperament, environmental stressors, and family dynamics is needed to better understand the complex, reciprocal processes underlying emotion socialization.

While the present study offers both practical and theoretical contributions, its findings should be interpreted with caution due to several limitations. First, the exclusive reliance on self-report measures may introduce response biases; parents might underreport negative behaviors or overestimate supportive practices, which could affect the observed associations. In addition, the CCNES has not been formally validated in Greek, and results should be interpreted in light of potential cultural or linguistic nuances that may affect response accuracy. The cross-sectional design further limits our ability to infer causality—although the correlations and regression analyses provide a snapshot of current parental practices and child outcomes, longitudinal research is needed to clarify the directionality and stability of these relationships. Moreover, the use of a non-probability sample further limits representativeness, which restricts the generalizability of our findings to the broader population of Greek families. Furthermore, detailed socio-demographic data (e.g., parental education, employment status, financial situation) and family structure information (e.g., number of siblings, birth order) were not collected, limiting our ability to explore contextual influences on parenting and child outcomes. Finally, residual confounding may be present; for example, variables such as parental age and socio-economic characteristics (e.g., educational attainment) were not adjusted for in our regression models, even though these factors could influence both parenting behaviors and children’s psychosocial outcomes. Future research should incorporate these variables to more accurately isolate the associations between parental emotion socialization and child adjustment.

Looking forward, several avenues for future research emerge. Longitudinal studies are needed to track how changes in parental behavior over time influence children’s emotional development and psychosocial adjustment. Such research could clarify whether reducing non-supportive responses leads to sustained improvements in child outcomes. Additionally, expanding the sample to include more diverse socio-economic and regional groups within Greece—and even cross-cultural comparisons—would enhance our understanding of how universal versus culturally specific these processes might be. Further research could also integrate qualitative methods to capture the nuanced experiences of parents and children, offering a richer context for interpreting quantitative findings.

Moreover, exploring potential mediating mechanisms could deepen our insight into how parental emotion socialization exerts its influence. For instance, future studies might investigate whether factors such as parental stress, child temperament, or the quality of parent–child attachment serve as mediators in the relationship between parenting practices and child outcomes [48].

In addition to psychosocial and behavioral outcomes, future research could explore the biological relevance of early emotion socialization. Emerging evidence suggests that parenting practices may influence neurobiological systems involved in emotion regulation and stress response, such as the hypothalamic–pituitary–adrenal (HPA) axis and neural circuits related to emotional processing. Investigating biological markers—such as cortisol levels or functional brain imaging—alongside parental responses and child outcomes could offer a more comprehensive understanding of the mechanisms linking early family interactions to long-term mental health. Integrating such measures would help clarify whether the patterns observed behaviorally also manifest at the neurobiological level [49,50,51].

In conclusion, the study underscores the critical role of parental emotion socialization in shaping children’s developmental trajectories. By replicating established associations between non-supportive parenting and child maladjustment in a Greek cultural context, this research provides important cross-cultural validation of theoretical models developed largely in Western settings. These findings highlight both the universal risk associated with punitive and dismissive parenting, and the need to tailor interventions to cultural values and family structures. As such, the study makes a valuable contribution to cross-cultural psychology and underscores the importance of culturally sensitive approaches in developmental research.

## 5. Conclusions

This study highlights the strong association between non-supportive parental responses—especially punitive and dismissive behaviors—and increased internalizing and externalizing problems in children. Supportive responses showed positive links to social competence but had less predictive power in the presence of negative parenting. These findings underscore the need for culturally sensitive interventions that reduce non-supportive parenting and promote emotional guidance. Despite limitations such as a cross-sectional design and self-report measures, the study contributes cross-cultural evidence on parenting and child adjustment in Greek families. Longitudinal and more diverse studies are needed to inform future interventions. While grounded in the specific cultural and familial dynamics of Greek society, these findings may offer broader relevance for other collectivistic or hierarchical cultural contexts, though further cross-cultural validation is necessary.

## Figures and Tables

**Table 1:** Demographic characteristics of children and parents.

Characteristic	Category	N	Percentage (%)
Child Gender	Boys	54	55.7%
	Girls	43	44.3%
Nationality/Ethnicity	Greek	100	100.0%
School Grade	Kindergarten (5 years)	2	2.0%
	Grade 1 (6 years)	2	2.0%
	Grade 2 (7 years)	8	8.0%
	Grade 3 (8 years)	11	11.0%
	Grade 4 (9 years)	23	23.0%
	Grade 5 (10 years)	23	23.0%
	Grade 6 (11 years)	30	30.0%
Parent Gender	Male	17	17.9%
	Female	78	82.1%
Child Age (years)	Mean (SD)	10.0 (1.7)	

**Table 2:** Descriptive summary of child behavior problems and parental responses.

Scale/Dimension	Min	Max	Mean (SD)	Median (Interquartile Range)
CBCL Scales				
Anxiety/Depression	0.00	12.00	3.05 (2.95)	2 (1–5)
Withdrawn/Depressed	0.00	9.00	1.45 (1.95)	1 (0–2)
Somatic Complaints	0.00	9.00	0.95 (1.72)	0 (0–1)
Internalizing Problems	0.00	25.00	5.33 (5.35)	3 (1–8)
Social Problems	0.00	12.00	1.84 (2.27)	1 (0–3)
Thought Problems	0.00	8.00	1.18 (1.61)	1 (0–2)
Attention Problems	0.00	13.00	2.49 (2.76)	2 (1–3)
Rule-Breaking Behavior	0.00	8.00	1.12 (1.49)	1 (0–2)
Aggressive Behavior	0.00	12.00	3.27 (3.08)	3 (1–5)
Externalizing Problems	0.00	16.00	4.05 (3.72)	3 (1–6)
Other Problems	0.00	8.00	2.22 (2.08)	2 (0–3)
Total Problems	0.00	56.00	22.55 (4.29)	22 (20–25)
Activities	2.70	14.00	8.61 (2.60)	8.7 (6.7–10.5)
Social Competence	3.30	12.50	8.02 (1.94)	8 (6.5–9.3)
School Competence	2.00	6.20	5.41 (0.78)	5.8 (5–6)
CCNES Dimensions				
Distress/Anxiety Response	1.50	5.08	2.93 (0.68)	2.92 (2.5–3.42)
Punitive Response	1.00	3.92	2.04 (0.63)	1.92 (1.67–2.33)
Expressive Encouragement Response	1.00	7.00	5.05 (1.33)	5.33 (4.58–5.83)
Emotion-Focused Response	1.00	7.00	5.53 (1.18)	5.75 (5.25–6.29)
Problem-Focused Response	3.17	7.00	5.90 (0.82)	5.96 (5.5–6.5)
Minimization/Devaluation Response	1.00	6.08	3.07 (1.15)	2.96 (2.21–3.67)

**Table 3:** Associations between children’s behavior problems and parental emotion socialization.

CBCL/CCNES Scale	Distress/Anxiety	Punitive	Expressive Encouragement	Emotion-Focused	Problem-Focused	Minimization/Devaluation
Anxiety/Depression	0.22 *	0.11	−0.09	0.03	0.07	0.32 **
Withdrawn/Depressed	0.11	0.14	−0.06	0.28 *	0.23 *	0.27 *
Somatic Complaints	0.20	0.05	0.02	−0.08	−0.06	0.30 **
Internalizing Problems	0.25 *	0.15	−0.07	0.05	0.06	0.34 **
Social Problems	0.25 *	0.24	−0.13	0.01	0.09	0.32 **
Thought Problems	0.16	0.16	−0.05	0.12	0.09	0.21
Attention Problems	0.15	0.02	0.03	0.16	0.05	0.06
Rule-Breaking Behavior	0.11	0.19	−0.15	0.02	0.02	0.08
Aggressive Behavior	0.08	0.37 **	−0.09	0.09	0.07	0.28 **
Externalizing Problems	0.06	0.34 **	−0.19	0.05	0.05	0.27 *
Other Problems	0.19	0.21	−0.08	0.04	0.04	0.20
Total Problems	0.25	0.29 **	−0.09	0.11	0.12	0.36 **
Activities	−0.08	−0.12	0.26 *	−0.06	0.11	−0.16
Social Competence	−0.03	0.01	0.20	0.14	0.20	0.02
School Competence	−0.05	−0.06	0.10	0.12	0.00	−0.02
Overall Competence	−0.11	−0.09	0.27 *	0.04	0.25 *	−0.13

Note: Spearman’s rho correlations were computed to assess the associations between CBCL and CCNES dimensions. Significance levels are indicated as * *p* < 0.05 and ** *p* < 0.01.

**Table 4:** Regression analysis of child and parent demographics on parental emotion socialization responses.

Dependent Variable	Predictor	β	SE	Standardized Beta	*t*	*p*
Distress/Anxiety Response	Child Gender (Girls vs. Boys)	0.019	0.024	0.091	0.80	0.426
	Child Age	0.000	0.007	−0.005	−0.043	0.966
	Parent Gender (Females vs. Males)	0.023	0.032	0.082	0.711	0.479
Punitive Response	Child Gender	−0.045	0.028	−0.176	−1.623	0.109
	Child Age	0.003	0.008	0.041	0.373	0.710
	Parent Gender	0.067	0.038	0.198	1.794	0.077
Expressive Encouragement Response	Child Gender	0.036	0.037	0.109	0.968	0.336
	Child Age	0.003	0.011	0.033	0.291	0.772
	Parent Gender	−0.023	0.049	−0.053	−0.461	0.646
Emotion-Focused Response	Child Gender	0.038	0.031	0.134	1.213	0.229
	Child Age	0.010	0.009	0.124	1.100	0.275
	Parent Gender	0.053	0.042	0.143	1.266	0.209
Problem-Focused Response	Child Gender	0.018	0.016	0.127	1.147	0.255
	Child Age	0.004	0.005	0.084	0.740	0.462
	Parent Gender	0.020	0.021	0.107	0.942	0.349
Minimization/Devaluation Response	Child Gender	−0.027	0.035	−0.084	−0.792	0.431
	Child Age	0.009	0.011	0.093	0.855	0.395
	Parent Gender	0.132	0.046	0.308	2.850	0.006

Note: Regression analyses utilized logarithmic transformations of the dependent variables.

**Table 5:** Regression models predicting child behavioral problems from parental emotion socialization and demographic factors.

Dependent Variable	Predictor	β	SE	Standardized Beta	*t*	*p*
Internalizing Problems	Child Gender (Girls vs. Boys)	0.149	0.087	0.188	1.712	0.091
	Child Age	0.023	0.026	0.098	0.904	0.369
	Parent Gender (Females vs. Males)	−0.061	0.119	−0.060	−0.519	0.606
	Distress/Anxiety Response	0.093	0.066	0.161	1.414	0.162
	Punitive Response	−0.026	0.080	−0.042	−0.327	0.745
	Expressive Encouragement Response	0.005	0.041	0.017	0.128	0.899
	Emotion-Focused Response	−0.025	0.051	−0.076	−0.497	0.621
	Problem-Focused Response	0.043	0.076	0.091	0.567	0.573
	Minimization/Devaluation Response	0.135	0.051	0.396	2.677	0.009
Externalizing Problems	Child Gender (Girls vs. Boys)	−0.133	0.074	−0.192	−1.803	0.076
	Child Age	−0.026	0.022	−0.126	−1.201	0.234
	Parent Gender (Females vs. Males)	0.053	0.101	0.059	0.527	0.600
	Distress/Anxiety Response	−0.003	0.056	−0.006	−0.054	0.957
	Punitive Response	0.206	0.068	0.383	3.041	0.003
	Expressive Encouragement Response	−0.023	0.043	−0.078	−0.546	0.587
	Emotion-Focused Response	0.039	0.043	0.135	0.911	0.365
	Problem-Focused Response	0.041	0.065	0.100	0.640	0.524
	Minimization/Devaluation Response	0.070	0.035	0.264	2.015	0.048
Total Problems	Child Gender (Girls vs. Boys)	−0.011	0.084	−0.014	−0.128	0.899
	Child Age	−0.013	0.025	−0.058	−0.527	0.600
	Parent Gender (Females vs. Males)	0.003	0.114	0.003	0.027	0.979
	Distress/Anxiety Response	0.099	0.063	0.181	1.571	0.121
	Punitive Response	0.186	0.063	0.304	2.934	0.004
	Expressive Encouragement Response	−0.033	0.039	−0.116	−0.850	0.398
	Emotion-Focused Response	0.008	0.049	0.025	0.165	0.869
	Problem-Focused Response	0.081	0.073	0.180	1.107	0.272
	Minimization/Devaluation Response	0.108	0.042	0.320	2.573	0.012

Note: For the regression analyses, logarithmic transformations were applied to the dependent variables.

## Data Availability

The data presented in this study are available on request from the corresponding author due to privacy reasons.

## Abbreviations

The following abbreviations are used in this manuscript:

CBCL	Child Behavior Checklist
CCNES	Coping with Children’s Negative Emotions Scale
